# Feasibility of Postpartum Blood Pressure Monitoring for Hypertensive Disorders in a Low-Resource Setting

**DOI:** 10.1016/j.jacadv.2025.101739

**Published:** 2025-05-28

**Authors:** Zainab Mahmoud, Chukwuebuka F. Okoye, Adaego A. Orji, Friday O. Ameh, Cecilia Nartey, Godwin Akaba, Bissallah A. Ekele, Erica L. Jamro, Kathryn J. Lindley, Dike B. Ojji, Mark D. Huffman

**Affiliations:** a Cardiovascular Division and Global Health Center, Department of Medicine, Washington University in St. Louis, St. Louis, Missouri, USA; b Cardiovascular Research Unit, University of Abuja and University of Abuja Teaching Hospital, Abuja, Nigeria; c Faculty of Clinical Sciences, University of Abuja, Abuja, Nigeria; d Division of Cardiology, Department of Medicine, Vanderbilt University Medical Center, Nashville, Tennessee, USA; e Department of Obstetrics and Gynecology, Vanderbilt University Medical Center, Nashville, Tennessee, USA; f The George Institute for Global Health, Sydney, Australia

**Keywords:** hypertension, hypertensive disorders of pregnancy, postpartum blood pressure monitoring

## Abstract

**Background:**

Hypertensive disorders of pregnancy (HDP) pose significant risks to maternal health globally, especially in Nigeria, which has the highest maternal mortality rate. Home blood pressure (BP) monitoring is a promising approach for managing HDP.

**Objectives:**

This study assessed the feasibility of a postpartum home BP-monitoring program for women with HDP in low-resource settings like Nigeria, focusing on recruitment, retention, and fidelity to monitoring protocols. HDP diagnoses associated with persistent hypertension were also assessed.

**Methods:**

Participants with HDP were enrolled into a prospective cohort. They were educated on BP monitoring and transmitted daily BP readings for 2 weeks. A control group of healthy postpartum women was also enrolled. The 12-week study involved assessments at prespecified intervals.

**Results:**

The study met its target of 90 participants (mean age: 30 years) and had high fidelity (96%) to daily BP recordings and retention (94%) at 12 weeks. The mean systolic BP decreased from 137 mm Hg to 125 mm Hg, and the mean diastolic BP decreased from 89 mm Hg to 84 mm Hg. During the initial 2-week period, 81.1% of normotensive participants experienced elevated BP, with 86.5% showing elevated BP over 12 weeks. In addition, 22% reported adverse cardiovascular events.

**Conclusions:**

The study demonstrates the feasibility of a postpartum BP-monitoring program in a low-resource setting like Nigeria, with high recruitment, fidelity, and retention. Continued monitoring beyond the immediate postpartum period is essential for improving outcomes. Further research is needed to evaluate the long-term effectiveness and scalability of such programs.

Cardiovascular (CV) disease is one of the leading causes of maternal mortality globally, and Nigeria has the highest burden of maternal deaths worldwide.^1^^,^^2^ Approximately 10% of all pregnancies are affected by hypertensive disorders of pregnancy (HDP) which include chronic hypertension, gestational hypertension, preeclampsia, eclampsia, hemolysis, elevated liver enzymes, and low platelet levels (HELLP) syndrome, and preeclampsia superimposed on chronic hypertension.^3^ Approximately 32% of maternal deaths in Nigeria are due to HDP.^4^ Most deaths are preventable with timely implementation of evidence-based strategies.^5^

Most maternal deaths also occur postpartum, a crucial transition phase for maternal care as it shifts from obstetricians to primary care providers and other specialists.^6^^,^^7^ This postpartum period, often referred to as the 4th trimester, is a critical time for monitoring and treating women at the highest risk of CV complications including cardiomyopathy, stroke, and eclampsia. Enhanced risk stratification, early detection, and comprehensive CV treatment during this postpartum period could reduce these complications.^5^ Postpartum women have significant fluctuations in blood pressure (BP) in the weeks following delivery, with peak systolic BP occurring at a median of 8 (IQR: 5-15) days postpartum, when patients are home and often unmonitored.^8^ These BP spikes are associated with hypertensive crises and increased risk of stroke and heart failure.^9^^,^^10^

In 2018, the American College of Obstetricians' Presidential Taskforce on Redefining Postpartum Care released recommendations for postpartum care, including the management of HDP. These recommendations emphasized the importance of evaluating BP within specific timeframes after childbirth. Patients with severe hypertension should have their BP evaluated within 72 hours, while those with nonsevere hypertension should have it assessed no later than 7 to 10 days postpartum.^11^ Postpartum home BP-monitoring program is an effective strategy to adhere to this recommendation and have been increasingly implemented in high-income countries.^12^, ^13^, ^14^, ^15^, ^16^, ^17^, ^18^ However, in low- and middle-income countries like Nigeria, home BP monitoring among patients with HDP is limited. A 2021 nationwide study examining adherence to postpartum recommendations in 366 women with HDP in tertiary hospitals in Nigeria showed that only 37% of women had a BP check within 3 to 5 days, highlighting a significant gap in utilizing postpartum BP monitoring and treatment.^19^

Emerging US data suggest promising outcomes for postpartum BP-monitoring programs compared to usual care. For example, these programs increase postpartum BP ascertainment (92% vs 44%), improve postpartum visit attendance (69% vs 58%; adjusted OR: 2.30 [95% CI: 1.05-5.07], *P* = 0.04), decrease postpartum readmissions (1.2% vs 2.2%; OR: 0.5 [95% CI: 0.26-1.04]; *P* = 0.06), and enhance transitions of care from obstetricians to cardiologists (79% vs 71%; OR: 1.50 [95% CI: 1.22-1.93]).^12^^,^^20^ A similar program in the United Kingdom has also demonstrated higher BP control among patients who participate in home BP monitoring than with usual care (between-group difference in 24-hour mean systolic BP of −6.51 mm Hg (95% CI: −8.80 to −4.22; *P* < 0.001) and diastolic BP of −5.80 mm Hg (95% CI: −7.40 to −4.20; *P* < 0.001).^21^

These results highlight the potential benefits of implementing postpartum BP-monitoring programs. However, it is important to generate contextually relevant data, especially in low- and middle-income countries (LMICs) like Nigeria, where the burden is high and implementation challenges may differ. The objective of this study was to contextualize and evaluate the feasibility of postpartum BP monitoring in patients with HDP in Abuja, Nigeria. In addition, we conducted exploratory analyses of BP trends by HDP diagnosis. The insights gained from this study could provide a template for potential expansion of the program to larger studies that can assess implementation and effectiveness of postpartum BP-monitoring program in improving postpartum BP control and CV outcomes among patients with HDP in Nigeria and other low-resource settings.

## Methods

### Study design, setting, and procedures

This pilot study, using a prospective cohort design, aimed to examine the feasibility of a BP-monitoring program for patients diagnosed with HDP. The study protocol was reviewed and approved by the ethics board at the University of Abuja Teaching Hospital (UATH), Federal Capital Territory, Nigeria and the institutional review board at Washington University of St. Louis. Informed consent was obtained from each participant prior to enrolment.

The study enrolled adult participants aged over 18 years who had delivered at the UATH and received a diagnosis of any form of HDP. Adult participants aged over 18 years who had also delivered at UATH but had no diagnosis of HDP were enrolled in a control arm. Recruitment took place between October 2022 and March 2023, with participants voluntarily enrolled from the antenatal, labor, or postnatal wards. The BP-monitoring program spanned 12 weeks, with the initial 2 weeks involving daily home BP measurements (Central Illustration). Participants were educated by trained research team members on how to measure their own BP using an automated BP monitor (Omron series 3, OMRON Healthcare). They were observed by research assistants while taking their BP and were instructed to transmit their readings to the study team through text messaging daily. Participants were encouraged to take BP readings at consistent times each day, preferably in the morning and/or evening. Measurements followed a standard protocol recommended by the American Heart Association.^22^ However, for practical reasons, in a postpartum population with competing demands and in a low-resource setting, strict timing was not enforced. The participants were also educated on abnormal parameters as well as concerning symptoms to monitor and report. In addition, participants received a reference booklet, which included a diary for recording their BP readings. This tool was adapted from the VERONICA (deliVERy of optimal blood pressure coNtrol in afrICA)-Nigeria study (Supplement A).^23^ To enhance adherence to study procedures, participants received daily text message reminders during the initial 2-week period. In addition, the study team communicated with participants at 2-week, 6-week, and 12-week follow-up phone visits to assess for any CV symptoms or complications using a structured questionnaire. During these follow-up phone visits, a management protocol was implemented to guide the advice provided to participants with abnormal BP readings (Supplement B). Participants were considered lost to follow-up if the study team was unable to reach them via 2 telephone numbers on at least 3 occasions during the 12-week follow-up period.

**Central Illustration:**
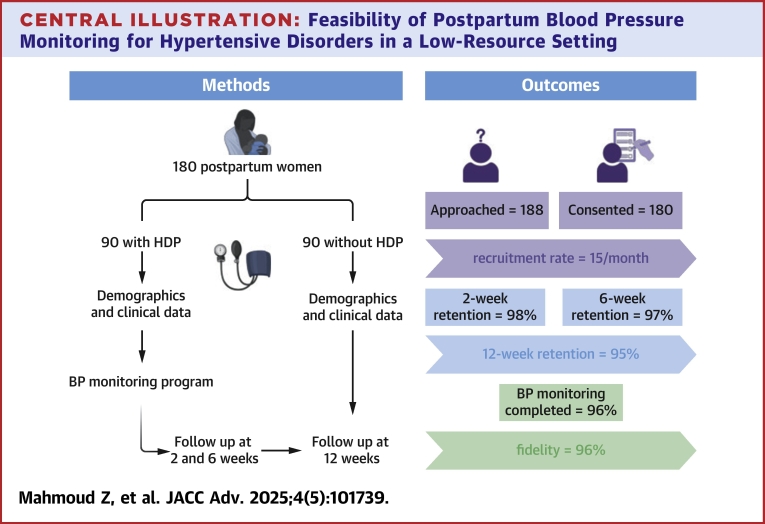
Feasibility of Postpartum Blood Pressure Monitoring for Hypertensive Disorders in a Low-Resource Setting

### Study outcomes and data analysis

The primary feasibility outcome was the recruitment rate, measured by the number of participants recruited each month with a predefined target of 15 participants per month over 6 months. Secondary outcomes included fidelity and retention. Fidelity was defined as adherence to study procedures, with a predefined fidelity threshold of a minimum of 10 daily home BP recordings per participant throughout the 2-week BP-monitoring phase (recordings measured and transmitted >71% of eligible days). Participant retention within the home BP-monitoring program was defined by the presence of a recorded BP reading on either day 13 or day 14. Feasibility outcomes were selected to ensure sufficient data were available for analysis while considering the challenges postpartum participants face in low-resource settings. The predefined fidelity threshold of 10 out of 14 readings was set as the minimum for adherence, which allowed us to evaluate fidelity while accounting for potential challenges in daily monitoring. Notably, in implementation studies, fidelity thresholds vary widely, from 60% to 98%.^24^ Other predefined exploratory and safety outcomes were the detection of elevated BP >140/90 mm Hg and incidence of adverse CV events, including CV hospital readmission, postpartum preeclampsia, hypertensive urgency/emergency, stroke, heart failure/pulmonary edema, seizure, or CV mortality during the 12-week postpartum period. All-cause mortality was also assessed. Temporal trends in systolic blood pressure (SBP) and diastolic blood pressure (DBP) were assessed overall and by HDP diagnosis utilizing Spearman correlations, and differences in SBP and DBP across HDP diagnoses were evaluated using ANOVA.

Demographic information and co-morbidities of participants were summarized using descriptive statistics. Descriptive data were reported as mean ± SD, and median (IQR) for continuous variables if data were skewed. The recruitment rate was determined by calculating the mean number of participants enrolled each month throughout the study duration. Fidelity and retention were calculated as the proportion of participants who achieved the thresholds as indicated earlier. Continuous variables were compared using Student's *t*-test and Mann–Whitney *U* test, while categorical variables were assessed using chi-squared test. A two-sided *P* value < 0.05 was used to define statistical significance without adjustment for multiple testing. In exploratory analysis, a logistic regression model was used to evaluate associations between patient demographics and HDP diagnosis with persistent hypertension at 12 weeks using a threshold of 140/90 mm Hg and 130/80 mm Hg according to the American College of Obstetricians and Gynecologists and American Heart Association guidelines, respectively.^25^^,^^26^ Adjustment factors included in the final model regardless of statistical significance were selected a priori and comprised age and anti-hypertensive medication use at 12 weeks, based on their established clinical relevance.^27^, ^28^, ^29^ All analyses were performed using a complete case approach. SAS program (version 9.4) was used for statistical analyses. The study findings were presented in accordance with the Strengthening the Reporting of Observational Studies in Epidemiology (STROBE) guidelines.^30^

## Results

From October 2022 to March 2023, 90 out of 92 eligible participants with HDP who were approached were successfully enrolled in the postpartum BP-monitoring program, and 90 out of 96 eligible participants without HDP who were approached were enrolled in the control arm (Supplemental Figure 1). The overall median age was 30 years (IQR: 26-37 years). Among participants with HDP, 62% attained education beyond high school, and 43% had no source of income. Regarding HDP diagnoses, 25% had a diagnosis of preeclampsia, 9% had gestational hypertension, 8% had chronic hypertension with superimposed preeclampsia, 5% had eclampsia/HELLP, and 3% had chronic hypertension. Notably, 73% of participants had no known co-morbidities, 29% were primiparous, 20% were primigravida, and 1% reported the use of aspirin for primary prophylaxis against preeclampsia (Table 1).

**Table 1:** Baseline Demographic and Clinical Characteristics Among Participants With Hypertensive Disorders of Pregnancy, by Diagnosis

	Overall (N = 90)^a^	Chronic HTN + Chronic HTN/ Preeclampsia (n = 19)	Gestational (n = 17)	Preeclampsia + Eclampsia/HELLP (n = 54)	*P* Value
Demographic characteristics					
Age (y)	30 (26-37)	37 (30-38)	32 (28-35)	29 (24-35)	0.003
Education					0.36
None	2 (2.2)	0 (0.0)	0 (0.0)	2 (3.7)	
Primary/Secondary school	32 (35.6)	5 (26.3)	4 (23.5)	23 (42.6)	
Diploma/Undergraduate/Postgraduate	56 (62.2)	14 (73.7)	13 (76.5)	29 (53.7)	
Ethnicity					0.80
Hausa	15 (16.7)	3 (15.8)	3 (17.6)	9 (16.7)	
Igbo	14 (15.6)	2 (10.5)	5 (29.4)	7 (13.0)	
Yoruba	9 (10.0)	2 (10.5)	1 (5.9)	6 (11.1)	
Other	52 (57.8)	12 (63.2)	8 (47.1)	32 (59.3)	
Employment status					0.0003
Full time	25 (27.8)	6 (31.6)	9 (52.9)	10 (18.5)	
Part time	3 (3.3)	0 (0.0)	2 (11.8)	1 (1.9)	
Student	3 (3.3)	0 (0.0)	0 (0.0)	3 (5.6)	
Homemaker	34 (37.8)	2 (10.5)	5 (29.4)	27 (50.0)	
Unemployed	25 (27.8)	11 (57.9)	1 (5.9)	13 (24.1)	
Individual monthly income (Naira)					0.08
No income	39 (43.3)	5 (26.3)	5 (29.4)	29 (53.7)	
<9,000	5 (5.6)	2 (10.5)	0 (0.0)	3 (5.6)	
9,000-29,000	20 (22.2)	4 (21.1)	5 (29.4)	11 (20.4)	
30,000-100,000	23 (25.6)	8 (42.1)	7 (41.2)	8 (14.8)	
>100,000	3 (3.3)	0 (0.0)	0 (0.0)	3 (5.6)	
Baseline clinical characteristics					
Comorbidities					
Any comorbidity present^b^	24 (26.7)	8 (42.1)	2 (11.8)	14 (25.9)	0.14
Diabetes (type 2)	3 (3.3)	1 (5.3)	0 (0.0)	2 (3.7)	1.00
Obesity/overweight	17 (18.9)	8 (42.1)	2 (11.8)	7 (13.0)	0.02
Aspirin use	1 (1.1)	0 (0.0)	0 (0.0)	1 (1.9)	1.00
Multiple gestation	6 (6.7)	0 (0.0)	1 (5.9)	5 (9.3)	0.43
Cesarean delivery	66 (73.3)	14 (73.7)	11 (64.7)	41 (75.9)	0.64
Primiparous	26 (28.9)	4 (21.1)	2 (11.8)	20 (37.0)	0.10
Primigravid	18 (20.0)	1 (5.3)	1 (5.9)	16 (29.6)	0.02
Anti-hypertensive medication(s) at baseline	73 (81.1)	19 (100.0)	12 (70.6)	42 (77.8)	0.03
Anti-hypertensive medication(s) at 12 wk	39 (45.9)	13 (72.2)	4 (23.5)	22 (44.0)	0.01

Values are median (IQR) or n (%).

HELLP = hemolysis, elevated liver enzymes, and low platelet levels; HTN = hypertension; SLE = systemic lupus erythematosus.

a Diagnosis of chronic hypertension with or without superimposed pre-eclampsia, gestational hypertension, pre-eclampsia, or eclampsia/HELLP.

b Comorbidities self-reported include blood clots/blood disorder (n = 1), depression/anxiety/mental illness (n = 1), diabetes type 2 (n = 3), heart failure (n = 1), hepatitis B (n = 4), obesity/overweight (n = 17), sickle cell disease (n = 1), sleep apnea (n = 1), and thyroid disease (n = 1). No participants reported asthma, atrial fibrillation, autoimmune disorder, cancer, diabetes type 1, heart attack/coronary artery disease, HIV, hepatitis C, hyperlipidemia, kidney disease, lupus/SLE, peripartum cardiomyopathy, seizure disorder, stroke, or any other condition.

The primary feasibility outcome measured by the rate of recruitment was a mean of 15 ± 6 participants with a diagnosis of HDP per month. The median number of BP measurements among participants was 14 (IQR: 13-14) daily BP recordings during the 2-week monitoring period, with 96% achieving the fidelity threshold of at least 10 BP measurements over the 2-week monitoring period. Participant retention rates at 2, 6, and 12 weeks were 98%, 97%, and 95%, respectively (Table 2).

**Table 2:** Feasibility Outcomes

Approached	188
Consented	180 (96%)
Recruitment rate per month	15 ± 6
Retention rate	N = 90
2 wk	88 (98%)
6 wk	87 (97%)
12 wk	85 (95%)
Fidelity to study procedures^a^	86 (96%)

Values are n, n (%), or mean ± SD.

a Fidelity defined as adherence to study procedures, with a predefined fidelity threshold of a minimum of 10 daily home BP recordings per participant throughout the 2-week BP -monitoring phase.

In exploratory analysis, mean SBP at enrollment and 12-week follow-up was 137 ± 20 mm Hg and 125 ± 17 mm Hg, respectively. Mean DBP at enrollment and 12-week follow-up was 89 ± 14 mm Hg and 85 ± 13 mm Hg, respectively (Table 3). Among participants with normal BP at enrollment, 81% had elevated BP (>140/90) on at least 1 day during the initial 2-week monitoring period, and 87% subsequently had elevated BP on at least 1 day during the 12-week monitoring period. Participants experienced elevated BP on a median of 15% of follow-up days (IQR: 8%-43%) and their initial episode of elevated BP at a median follow-up of 5.5 days (IQR: 3.0-10.0 days). At 12 weeks, 36% of participants who completed follow-up had elevated BP, and 41% were on antihypertensive medications. Among participants who had elevated BP at enrollment, 39% that completed follow-up had elevated BP at 12 weeks, and 60% were on antihypertensive medications at 12 weeks.

**Table 3:** Mean Systolic and Diastolic Blood Pressure Among Participants With Hypertensive Disorders of Pregnancy, by Study Participation Day

Day	Overall (N = 90)^a^	Chronic HTN + Chronic HTN/Preeclampsia (n = 19)	Gestational HTN (n = 17)	Preeclampsia + Eclampsia/HELLP (n = 54)	*P* Value^b^
Systolic blood pressure					
1	137.4 ± 19.8	136.6 ± 20.7	139.2 ± 19.6	137.1 ± 19.9	0.91
2	131.2 ± 16.5	132.5 ± 17.4	128.3 ± 13.2	131.6 ± 17.3	0.73
3	129.2 ± 15.3	129.8 ± 13.7	125.4 ± 15.1	130.2 ± 16.0	0.57
4	131.5 ± 15.4	130.8 ± 14.6	125.9 ± 12.9	133.6 ± 16.2	0.22
5	131.6 ± 15.0	132.9 ± 11.8	131.0 ± 15.0	131.3 ± 16.2	0.91
6	132.5 ± 14.6	137.5 ± 13.5	129.6 ± 12.6	131.6 ± 15.4	0.23
7	128.2 ± 13.0	133.3 ± 13.9	130.1 ± 12.9	125.6 ± 12.3	0.08
8	128.2 ± 14.6	134.1 ± 14.5	129.1 ± 13.3	125.9 ± 14.7	0.15
9	127.6 ± 12.1	130.0 ± 13.5	129.3 ± 13.8	126.1 ± 10.9	0.42
10	125.1 ± 12.0	129.0 ± 13.9	126.9 ± 12.5	123.1 ± 10.8	0.15
11	127.7 ± 15.2	133.6 ± 17.3	128.5 ± 15.0	125.3 ± 14.1	0.14
12	128.1 ± 14.1	132.3 ± 17.7	126.6 ± 11.0	127.2 ± 13.8	0.42
13	127.8 ± 13.3	136.8 ± 13.2	125.5 ± 12.3	125.5 ± 12.4	**0.01**
14	126.2 ± 14.6	132.2 ± 10.4	128.4 ± 15.0	123.1 ± 15.2	0.06
42	125.7 ± 15.1	129.2 ± 15.5	124.8 ± 12.0	124.5 ± 16.0	0.51
84	125.4 ± 16.5	132.3 ± 11.9	131.5 ± 19.4	120.0 ± 15.5	**0.01**
Correlation^c^	**r = −0.16, *P* < 0.0001**	r = −0.05, *P*= 0.40	r = −0.07, *P* = 0.24	**r = −0.25, *P* < 0.0001**	
Diastolic blood pressure					
1	89.4 ± 14.3	89.4 ± 15.6	90.8 ± 12.5	88.9 ± 14.5	0.89
2	87.1 ± 13.5	88.0 ± 13.8	83.9 ± 9.7	87.7 ± 14.4	0.59
3	86.0 ± 11.6	86.5 ± 12.1	82.1 ± 11.2	87.0 ± 11.5	0.34
4	88.3 ± 13.0	87.5 ± 11.2	82.6 ± 10.9	90.5 ± 13.8	0.11
5	88.5 ± 10.7	90.8 ± 7.9	87.6 ± 8.8	88.0 ± 12.1	0.61
6	89.1 ± 11.3	93.9 ± 8.3	87.9 ± 9.5	87.8 ± 12.5	0.13
7	87.0 ± 11.0	92.7 ± 9.9	85.7 ± 10.6	85.4 ± 11.1	**0.04**
8	88.2 ± 12.1	94.4 ± 9.8	86.9 ± 7.8	86.5 ± 13.5	0.07
9	87.1 ± 10.6	89.8 ± 10.5	87.9 ± 11.9	85.8 ± 10.2	0.35
10	85.7 ± 10.3	89.9 ± 10.6	84.8 ± 10.4	84.4 ± 10.0	0.14
11	87.4 ± 12.0	92.4 ± 13.2	86.4 ± 10.5	85.8 ± 11.7	0.13
12	88.2 ± 10.2	92.6 ± 11.0	86.2 ± 7.5	87.4 ± 10.4	0.14
13	88.4 ± 11.7	97.4 ± 12.5	84.5 ± 11.0	86.6 ± 10.1	**0.001**
14	86.6 ± 11.7	92.2 ± 10.5	86.4 ± 11.1	84.6 ± 11.9	0.055
42	84.6 ± 12.8	89.5 ± 13.5	81.8 ± 11.0	83.6 ± 12.9	0.15
84	85.2 ± 12.8	88.4 ± 9.9	89.8 ± 14.7	82.1 ± 12.7	0.09
Correlation^c^	**r = −0.07, *P* = 0.01**	r = −0.05, *P* = 0.41	r = −0.01, *P* = 0.91	**r = −0.13, *P* = 0.0002**	

Values are mean ± SD. **Bold** values indicate statistically significant differences, with *P* < 0.05.

HELLP = hemolysis, elevated liver enzymes, and low platelet levels; HTN = hypertension.

a Diagnosis of chronic hypertension with or without superimposed pre-eclampsia, gestational hypertension, pre-eclampsia, or eclampsia/HELLP.

b ANOVA comparison across diagnoses.

c Spearman correlation coefficient and *P* value.

**Figure 1:**
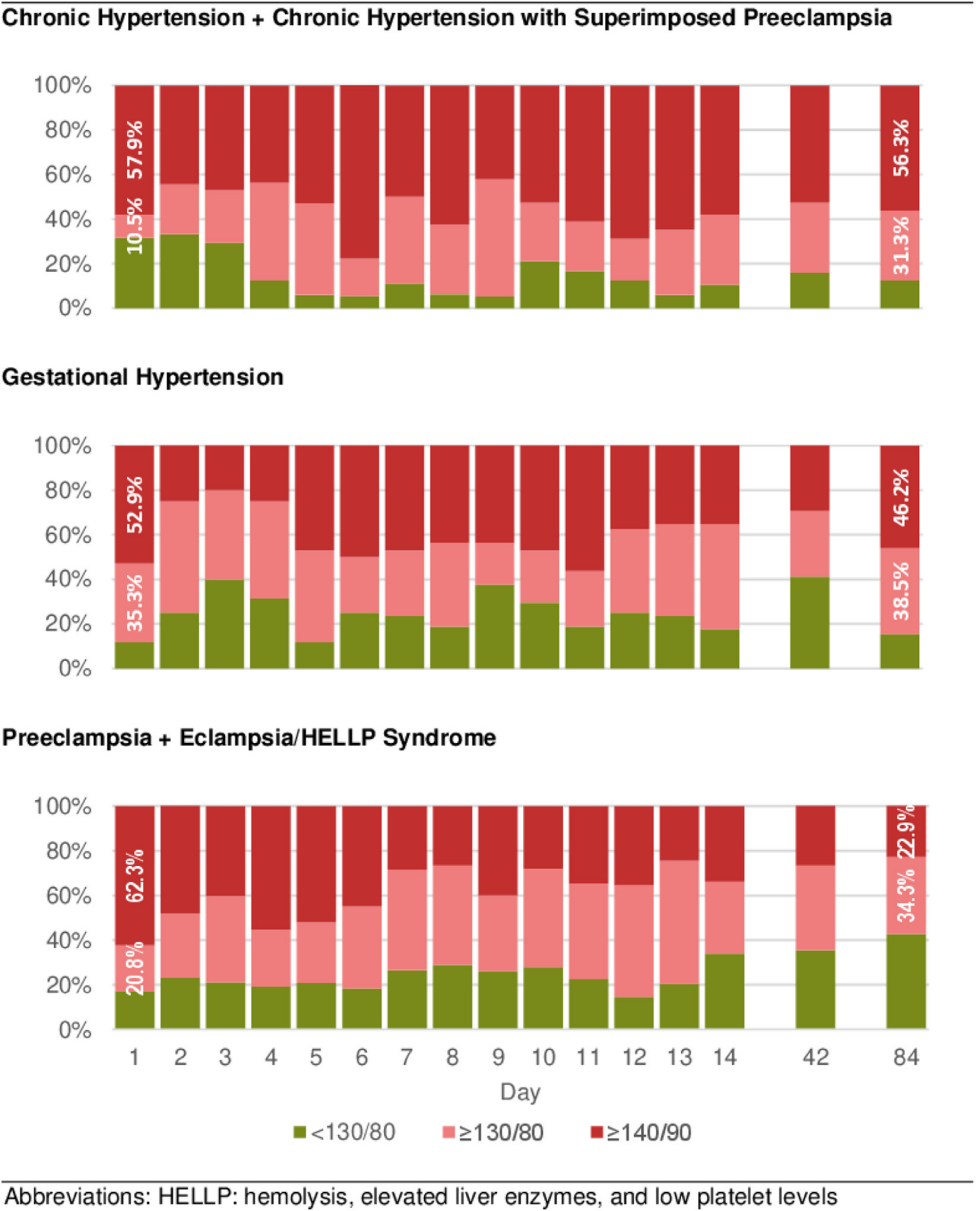
Participant Blood Pressure Control Over Time by Diagnosis

Postpartum BP control varied by diagnosis (Figure 1). Among participants with chronic hypertension or chronic hypertension with superimposed preeclampsia, 58% had elevated BP at enrollment, and 56% had uncontrolled BP (≥140/80 mm Hg) at 12-week follow-up. In those diagnosed with gestational hypertension, 53% had elevated BP at diagnosis, with 46% remaining uncontrolled at 12 weeks. For participants with preeclampsia, eclampsia, or HELLP syndrome, while 62% had elevated BP at enrollment, only 23% had uncontrolled BP at the 12-week follow-up.

Using diagnosis as a predictor of SBP at 12 weeks and adjusting for age and antihypertensive medication use, mean SBP was significantly lower in patients with preeclampsia/eclampsia/HELLP than in those with chronic hypertension (120.0 mm Hg; 95% CI: 115.0-125.0 vs 132.3 mm Hg; 95% CI: 124.8-139.7, *P* < 0.01). Mean SBP among participants with gestational hypertension and mean DBP across all diagnoses did not significantly differ from those of participants with chronic hypertension or chronic hypertension with superimposed preeclampsia (Table 4).

**Table 4:** Unadjusted and Adjusted Systolic and Diastolic Blood Pressure Estimates and Odds of High Blood Pressure at 12-Week Follow-Up Among Participants (n = 64) With Hypertensive Disorders of Pregnancy, by Diagnosis

	Unadjusted	*P* Value^a^	Adjusted^b^	*P* Value^a^
Mean systolic blood pressure, mm Hg				
Chronic HTN + chronic HTN/preeclampsia	132.3 (124.8-139.7)	-	104.5 (82.6-126.5)	-
Gestational HTN	131.5 (123.3-139.8)	0.90	110.3 (89.8-130.8)	0.31
Preeclampsia + Eclampsia/HELLP syndrome	120.0 (115.0-125.0)	**0.01**	99.1 (81.1-117.1)	0.25
Mean diastolic blood pressure, mm Hg				
Chronic HTN + Chronic HTN/Preeclampsia	88.4 (82.4-94.3)	-	65.6 (47.9-83.3)	-
Gestational HTN	89.8 (83.1-96.4)	0.76	71.8 (55.3-88.3)	0.18
Preeclampsia + Eclampsia/HELLP syndrome	82.1 (78.1-86.2)	0.09	64.6 (50.1-79.1)	0.80
High blood pressure (≥140/90 mm Hg)				
Chronic HTN + Chronic HTN/Preeclampsia	Ref	-	Ref	-
Gestational HTN	0.67 (0.15-2.90)	0.61	1.63 (0.27-9.83)	0.32
Preeclampsia + Eclampsia/HELLP syndrome	0.23 (0.07-0.82)	**0.02**	0.58 (0.13-2.64)	0.21
High blood pressure (≥130/80 mm Hg)				
Chronic HTN + Chronic HTN/Preeclampsia	Ref	-	Ref	-
Gestational HTN	0.79 (0.10-6.50)	0.50	1.97 (0.20-19.05)	0.20
Preeclampsia + Eclampsia/HELLP syndrome	0.19 (0.04-0.97)	**0.02**	0.37 (0.06-2.23)	0.055

Values are OR (95% CI). **Bold** values indicate statistically significant differences, with *P* < 0.05.

HELLP = hemolysis, elevated liver enzymes and low platelets; HTN = hypertension.

a Compared with chronic HTN + chronic HTN/Preeclampsia.

b Adjusted for age and anti-hypertensive medication status at 12-week follow-up.

Out of 85 participants in the BP-monitoring arm who successfully completed the 12-week follow-up, 15% reported experiencing an adverse CV event, with an additional 13% indicating the presence of at least one CV symptom. Out of 78 participants in the control arm who completed the 12-week follow-up, 13% reported at least one CV symptom, but none had any CV event (Supplemental Table 2).

## Discussion

This study demonstrates the feasibility of a postpartum BP-monitoring program in a tertiary hospital in Abuja, Nigeria. The study achieved its target recruitment rate and maintained high levels of fidelity and participant retention up to 12 weeks postpartum. The recruitment rate, fidelity, and 12-week retention were higher than those in similar studies in high-income countries.^16^^,^^21^ The study's success in enrollment and retention demonstrates the potential of utilizing home BP monitoring in patients with HDP in a low-resource setting. These findings offer valuable insights for potential expansion of BP monitoring to larger studies that can assess implementation and effectiveness of postpartum BP-monitoring program in improving postpartum BP control and CV outcomes among patients with HDP in Nigeria and possibly other LMICs.

Participants enrolled in this study were noted to be overall young with a median age of 30 years (IQR: 26-36 years). In addition, they were predominantly healthy at baseline, with 73% of participants having no known comorbidities. This is relevant because advanced maternal age (>35 years) and comorbidities are 2 recognized risk factors for HDP and form the basis for the preeclampsia-prevention guidelines.^31^^,^^32^ It also underscores the importance of addressing hypertension in pregnancy even among seemingly low-risk populations. These findings suggest that relying solely on traditional risk factors may cause missed opportunities for prevention in this population.

One significant contribution of this study is the long-term BP trend data provided for women with HDP. This study is one of the first to report BP trends in women with a diagnosis of HDP with BP readings at 12 weeks postpartum and the first, to our knowledge, in sub-Saharan Africa. It offers new insights into BP trends by HDP diagnosis and association with persistent hypertension. However, larger studies are needed to further characterize these trends as well as understand how to use these data to improve outcomes. For example, the POP-HT (Physician Optimized Postpartum Hypertension Treatment Trial) trial in the United Kingdom showed the benefits of a physician-guided remote telemonitoring program on enhancing postpartum BP control. However, one of the limitations of the study was that it was a predominantly non-Hispanic White population.^21^ In contrast, the current study was conducted in an African population who are predominantly of low socioeconomic status, providing a distinctive demographic perspective. In exploratory analyses, we found that participants with preeclampsia/eclampsia/HELLP had lower mean SBP than those with chronic hypertension. However, SBP among those with gestational hypertension and mean DBP across all diagnoses did not significantly differ from that among those with chronic hypertension or chronic hypertension with superimposed preeclampsia. These findings may be influenced by limited statistical power for subgroup analyses. As a pilot study, the sample size was not powered to detect differences in BP trends at 12 weeks postpartum. The results of the study also revealed important patterns in postpartum BP and the prevalence of elevated BP among study participants. Among those with normal BP at enrollment, 87% had elevated BP on at least 1 day during the 12-week monitoring period. These findings highlight the need for continued monitoring and management of BP in the postpartum period, even for those who appear normotensive at the outset.

No participants in the control group reported a CV event at 12 weeks compared to 22% in the HDP group indicating that these participants are at elevated risk of adverse events postpartum. This increased risk underscores the importance of monitoring and evidence-based BP-lowering interventions for this population. Implementing postpartum BP-monitoring programs could potentially mitigate these adverse events by allowing for earlier detection and better management of elevated BP and other CV risk factors.

## Strengths and limitations

Strengths of this study include its contribution of long-term BP trend data specific to women with HDP in a high-burden population, thus offering valuable insights into postpartum CV health. The successful implementation of a postpartum BP-monitoring program in a tertiary hospital in Nigeria demonstrates the feasibility of utilizing home BP monitoring to advance maternal CV care in a low-resource setting. The study achieved its targeted recruitment rate with high levels of fidelity and participant retention rates up to 12 weeks postpartum, surpassing comparable studies in high-income countries. Furthermore, the inclusion of an African population with predominantly low socioeconomic status provides a unique demographic perspective.

However, the study also has some limitations. While it presents novel data on postpartum BP trends in an African population, the sample size was relatively small, and larger studies are needed to further characterize these trends. In addition, the study was conducted in a tertiary hospital setting in Abuja, limiting the generalizability of findings to other healthcare settings or regions. CV symptoms and complications were self-reported and were not verified against medical charts, potentially leading to underestimation. However, data indicate that self-reporting of events is usually valid but may underestimate the true numbers. Furthermore, the study's short duration may not capture long-term CV outcomes, highlighting the need for extended follow-up in future investigations. This study did not include a cost analysis, which limits the understanding of the economics of scaling BP-monitoring programs. At the time of the study, the cost of a single BP monitor was approximately $50. While this represents an upfront expense, it may be cost-effective at the individual level when considering the higher costs associated with hospital readmission for a hypertensive emergency—typically exceeding $50, not including lost wages and caregiver burden. The widespread applicability of this intervention in LMICs may still be constrained by cost, particularly in resource-limited healthcare systems. Future studies will explore cost analyses to assess the economic feasibility and sustainability of such programs in LMICs such as Nigeria.

Despite these limitations, the study provides a foundation for understanding postpartum BP trends, risk factors for persistent hypertension, and feasibility of implementing BP-monitoring programs in resource-constrained settings. While findings may not be directly generalizable to the US due to differences in healthcare infrastructure and population demographics, they provide valuable insights for adapting home BP-monitoring programs globally, including in underserved US communities where maternal health disparities persist.

## Conclusions

This study demonstrated high recruitment, fidelity, and retention rates, highlighting the feasibility and potential utility of postpartum BP-monitoring programs among patients with HDP to improve maternal CV outcomes in low-resource settings. It underscores the importance of continued BP monitoring and targeted interventions for women with HDP. These findings lay the groundwork for larger studies to further evaluate and optimize postpartum care, ultimately aiming to reduce the high burden of maternal mortality and morbidity due to CV disease in Nigeria and similar contexts.

Perspectives **COMPETENCY IN SYSTEMS-BASED PRACTICE:** A postpartum home blood pressure–monitoring program for women with hypertensive disorders of pregnancy is feasible in a low-resource setting, as demonstrated in Abuja, Nigeria, with high recruitment, retention, and fidelity rates. The study highlights the need for blood pressure monitoring to continue beyond the immediate postpartum period due to the persistent risk of elevated blood pressure and adverse cardiovascular events. Implementing a home blood pressure–monitoring program postpartum could facilitate early detection and management of elevated blood pressure and potential adverse cardiovascular events in low-resource settings.

**TRANSLATIONAL OUTLOOK:** Further studies are necessary to evaluate the clinical effects of postpartum blood pressure monitoring in Nigeria and similar low-resource contexts.

## Funding support and author disclosures

This study was supported by grant funding from the American College of Cardiology, Association of Black Cardiologists, and Merck Foundation. The funder had no role in the design and conduct of the study; collection, management, analysis, and interpretation of the data; preparation, review, or approval of the manuscript; or decision to submit the manuscript for publication. Dr Mahmoud is supported by the 10.13039/100000050NIH/NHLBI grant K23HL173684. Dr Huffman has received travel support from the World Heart Federation and consulting fees from PwC Switzerland; has an appointment at The George Institute for Global Health, which has a patent and license and has received investment funding with the intent to commercialize fixed-dose combination therapy through its social enterprise business, George Medicines; and has pending patents for heart failure polypills. All other authors have reported that they have no relationships relevant to the contents of this paper to disclose.
