# Supplementary material for: Feasibility of Postpartum Blood Pressure Monitoring for Hypertensive Disorders in a Low-Resource Setting
Source: JACC Adv. 2025 May 28;4(5):101739. doi: 10.1016/j.jacadv.2025.101739 (PMC12235407; doi:10.1016/j.jacadv.2025.101739)
Supplement: Supplementary data [file mmc1.pdf]

Supplemental Table 1: Participants with High Blood Pressure, by Diagnosis and Study Participation Day

Day	Overall[a] N=90	Chronic + Chronic/Preeclampsia N=19	Gestational N=17	Preeclampsia + Eclampsia/HELLP N=54	P-Value[b]
**High Blood Pressure [n (%)] (≥140/90, mmHg)**					
1	53 (59.6%)	11 (57.9%)	9 (52.9%)	33 (62.3%)	0.78
2	37 (43.0%)	8 (44.4%)	4 (25.0%)	25 (48.1%)	0.26
3	32 (38.1%)	8 (47.1%)	3 (20.0%)	21 (40.4%)	0.25
4	37 (46.8%)	7 (43.8%)	4 (25.0%)	26 (55.3%)	0.11
5	42 (51.2%)	9 (52.9%)	8 (47.1%)	25 (52.1%)	0.93
6	44 (53.0%)	14 (77.8%)	8 (50.0%)	22 (44.9%)	0.06
7	31 (36.9%)	9 (50.0%)	8 (47.1%)	14 (28.6%)	0.17
8	29 (37.7%)	10 (62.5%)	7 (43.8%)	12 (26.7%)	**0.03**
9	35 (41.2%)	8 (42.1%)	7 (43.8%)	20 (40.0%)	0.96
10	32 (37.2%)	10 (52.6%)	8 (47.1%)	14 (28.0%)	0.11
11	37 (44.6%)	11 (61.1%)	9 (56.3%)	17 (34.7%)	0.09
12	34 (42.5%)	11 (68.8%)	6 (37.5%)	17 (35.4%)	0.06
13	29 (34.9%)	11 (64.7%)	6 (35.3%)	12 (24.5%)	**0.01**
14	34 (39.5%)	11 (57.9%)	6 (35.3%)	17 (34.0%)	0.18
42	27 (33.3%)	10 (52.6%)	5 (29.4%)	12 (26.7%)	0.12
84	23 (35.9%)	9 (56.3%)	6 (46.2%)	8 (22.9%)	**0.049**
**P-Value[c]:**	**0.02**	0.88	0.93	**0.0006**	
**High Blood Pressure [n (%)] (≥130/80, mmHg)**					
1	72 (80.9%)	13 (68.4%)	15 (88.2%)	44 (83.0%)	0.31
2	64 (74.4%)	12 (66.7%)	12 (75.0%)	40 (76.9%)	0.69
3	62 (73.8%)	12 (70.6%)	9 (60.0%)	41 (78.8%)	0.32
4	63 (79.7%)	14 (87.5%)	11 (68.8%)	38 (80.9%)	0.43
5	69 (84.1%)	16 (94.1%)	15 (88.2%)	38 (79.2%)	0.35
6	69 (83.1%)	17 (94.4%)	12 (75.0%)	40 (81.6%)	0.31
7	65 (77.4%)	16 (88.9%)	13 (76.5%)	36 (73.5%)	0.46
8	60 (77.9%)	15 (93.8%)	13 (81.3%)	32 (71.1%)	0.14

9	65 (76.5%)	18 (94.7%)	10 (62.5%)	37 (74.0%)	0.055
10	63 (73.3%)	15 (78.9%)	12 (70.6%)	36 (72.0%)	0.84
11	66 (79.5%)	15 (83.3%)	13 (81.3%)	38 (77.6%)	0.93
12	67 (83.8%)	14 (87.5%)	12 (75.0%)	41 (85.4%)	0.69
13	68 (81.9%)	16 (94.1%)	13 (76.5%)	39 (79.6%)	0.31
14	64 (74.4%)	17 (89.5%)	14 (82.4%)	33 (66.0%)	0.11
42	55 (67.9%)	16 (84.2%)	10 (58.8%)	29 (64.4%)	0.20
84	45 (70.3%)	14 (87.5%)	11 (84.6%)	20 (57.1%)	0.052
**P-Value[c]:**	**0.03**	0.49	0.99	**0.001**	

[a] Diagnosis of chronic hypertension with or without superimposed pre-eclampsia, gestational hypertension, pre-eclampsia, or eclampsia/HELLP

[b] Chi-square comparison across diagnoses

[c] Cochran-Armitage test for trend

Abbreviations: HELLP-hemolysis, elevated liver enzymes, and low platelet levels; SD- standard deviation

Supplemental Table 2: Cardiovascular Symptoms and Adverse Events at 12 Weeks Among Participants with HDP and Control Group Participants

	Participants with HDP (N=85)	Control (N=78)
**CV symptoms n (%)**	11 (13)	10 (13)
**CV event n (%)**	19 (22)	0
**Non-CV event, n (%)**	0	6 (8)
**None, n (%)**	55 (65)	62 (79)

Symptoms evaluated: chest pain, dyspnea, cough, palpitations, edema, orthopnea, paroxysmal nocturnal dyspnea, headache, visual changes, lightheadedness/syncope, seizure

CV events evaluated: CV hospital readmission, postpartum preeclampsia or eclampsia, hypertensive urgency/emergency, stroke, heart failure/pulmonary edema

Non-CV events evaluated: postpartum hemorrhage, infection, other event not listed

Abbreviations: CV: cardiovascular; HDP: hypertensive disorder of pregnancy

Supplemental Table 3: Baseline Demographic and Clinical Characteristics Among Participants with Hypertensive Disorders of Pregnancy and Control Group

	Overall [n=179]	HDP[a] [n=90]	Control [n=89]	P-Value
**Demographic Characteristics**				
Age (years), median (IQR)	30 (27, 35)	30 (26, 37)	30 (27, 33)	0.42
Education [n (%)]				0.92
None	5 (2.8%)	2 (2.2%)	3 (3.4%)	
Primary/ Secondary School	62 (34.6%)	32 (35.6%)	30 (33.7%)	
Diploma/Undergraduate/Postgraduate	112 (62.6%)	56 (62.2%)	56 (62.9%)	
Ethnicity [n (%)]				0.73
Hausa	25 (14.0%)	15 (16.7%)	10 (11.2%)	
Igbo	31 (17.3%)	14 (15.6%)	17 (19.1%)	
Yoruba	18 (10.1%)	9 (10.0%)	9 (10.1%)	
Other	105 (58.7%)	52 (57.8%)	53 (59.6%)	
Employment status [n (%)]				0.61
Full time	49 (27.4%)	25 (27.8%)	24 (27.0%)	
Part time	7 (3.9%)	3 (3.3%)	4 (4.5%)	
Student	11 (6.1%)	3 (3.3%)	8 (9.0%)	
Homemaker	65 (36.3%)	34 (37.8%)	31 (34.8%)	
Unemployed	47 (26.3%)	25 (27.8%)	22 (24.7%)	
Monthly income (Naira) [n (%)]				0.83
No income	74 (41.3%)	39 (43.3%)	35 (39.3%)	
<9,000	14 (7.8%)	5 (5.6%)	9 (10.1%)	
9,000-29,000	39 (21.8%)	20 (22.2%)	19 (21.3%)	
30,000-100,000	45 (25.1%)	23 (25.6%)	22 (24.7%)	
>100,000	7 (3.9%)	3 (3.3%)	4 (4.5%)	

Supplemental Table 3: Baseline Demographic and Clinical Characteristics Among Participants with Hypertensive Disorders of Pregnancy and Control Group

	Overall [n=179]	HDP[a] [n=90]	Control [n=89]	P-Value
**Demographic Characteristics**				
**Baseline Clinical Characteristics**				
Comorbidities [n (%)]				
Any Comorbidity Present[b]	33 (18.4%)	24 (26.7%)	9 (10.1%)	0.004
Diabetes (type2)	3 (1.7%)	3 (3.3%)	0 (0.0%)	0.25
Obesity/Overweight	21 (11.7%)	17 (18.9%)	4 (4.5%)	0.003
Aspirin use [n (%)]	1 (0.6%)	1 (1.1%)	0 (0.0%)	1.00
Multiple gestation [n (%)]	8 (4.5%)	6 (6.7%)	2 (2.2%)	0.28
Cesearean Delivery [n (%)]	78 (43.6%)	66 (73.3%)	12 (13.5%)	<.0001
Primiparous [n (%)]	54 (30.2%)	26 (28.9%)	28 (31.5%)	0.71
Primigravid [n (%)]	34 (19.0%)	18 (20.0%)	16 (18.0%)	0.73
Anti-hypertensive medication(s) at baseline [n (%)]	73 (40.8%)	73 (81.1%)	0 (0.0%)	<.0001
Anti-hypertensive medication(s) at 12-weeks [n (%)]	39 (24.2%)	39 (45.9%)	0 (0.0%)	<.0001

[a] Diagnosis of chronic hypertension with or without superimposed pre-eclampsia, gestational hypertension, pre-eclampsia, or eclampsia/HELLP

[b] Comorbidities self-reported include asthma, blood clots/blood disorder, depression/anxiety/mental illness, diabetes type 2, heart failure, hepatitis B or C, obesity/overweight, sickle cell disease, sleep apnea, and thyroid disease. No participants reported atrial fibrillation, autoimmune disorder, cancer, diabetes type 1, heart attack/coronary artery disease, HIV, hyperlipidemia, kidney disease, lupus/SLE, peripartum cardiomyopathy, seizure disorder, stroke, or any other condition. Abbreviations: IQR- Interquartile range; HELLP-hemolysis, elevated liver enzymes, and low platelet levels; HDP- hypertensive disorders of pregnancy

Supplemental Table 4. Unadjusted and Adjusted Systolic and Diastolic Blood Pressure Estimates and Odds of High Blood Pressure at 6-Week Follow-Up Among Participants (n=64) With Hypertensive Disorders of Pregnancy, By Diagnosis

	Unadjusted	P-Value[a]	Adjusted[b]	P-Value[a]
**Mean Systolic Blood Pressure**, mmHg (95% CI)				
Chronic + Chronic/Preeclampsia	129.2 (122.5, 135.9)	-	115.8 (95.1, 136.5)	-
Gestational	124.8 (117.7, 131.9)	0.38	112.5 (93.1, 131.9)	0.50
Preeclampsia + Eclampsia/HELLP	124.5 (120.2, 128.9)	0.25	113.4 (96.6, 130.3)	0.58
**Mean Diastolic Blood Pressure**, mmHg (95% CI)				
Chronic + Chronic/Preeclampsia	89.5 (83.9, 95.1)	-	75.5 (58.4, 92.7)	-
Gestational	81.8 (75.9, 87.7)	0.06	68.9 (52.9, 85.0)	0.11
Preeclampsia + Eclampsia/HELLP	83.6 (80.0, 87.3)	0.09	72.0 (58.1, 86.0)	0.33
**High Blood Pressure (≥140/90, mmHg)**, Odds Ratio (95% CI)				
Chronic + Chronic/Preeclampsia	Ref	-	Ref	-
Gestational	0.38 (0.10, 1.49)	0.48	0.43 (0.11, 1.77)	0.47
Preeclampsia + Eclampsia/HELLP	0.33 (0.11, 1.00)	0.20	0.45 (0.14, 1.51)	0.48
**High Blood Pressure (≥130/80, mmHg)**, Odds Ratio (95% CI)				
Chronic + Chronic/Preeclampsia	Ref	-	Ref	-
Gestational	0.27 (0.06, 1.28)	0.20	0.32 (0.06, 1.57)	0.18
Preeclampsia + Eclampsia/HELLP	0.34 (0.09, 1.35)	0.41	0.53 (0.12, 2.28)	0.90

[a] Compared with Chronic + Chronic/Preeclampsia

[b] Adjusted for age

Supplemental Figure 1: Study Participants

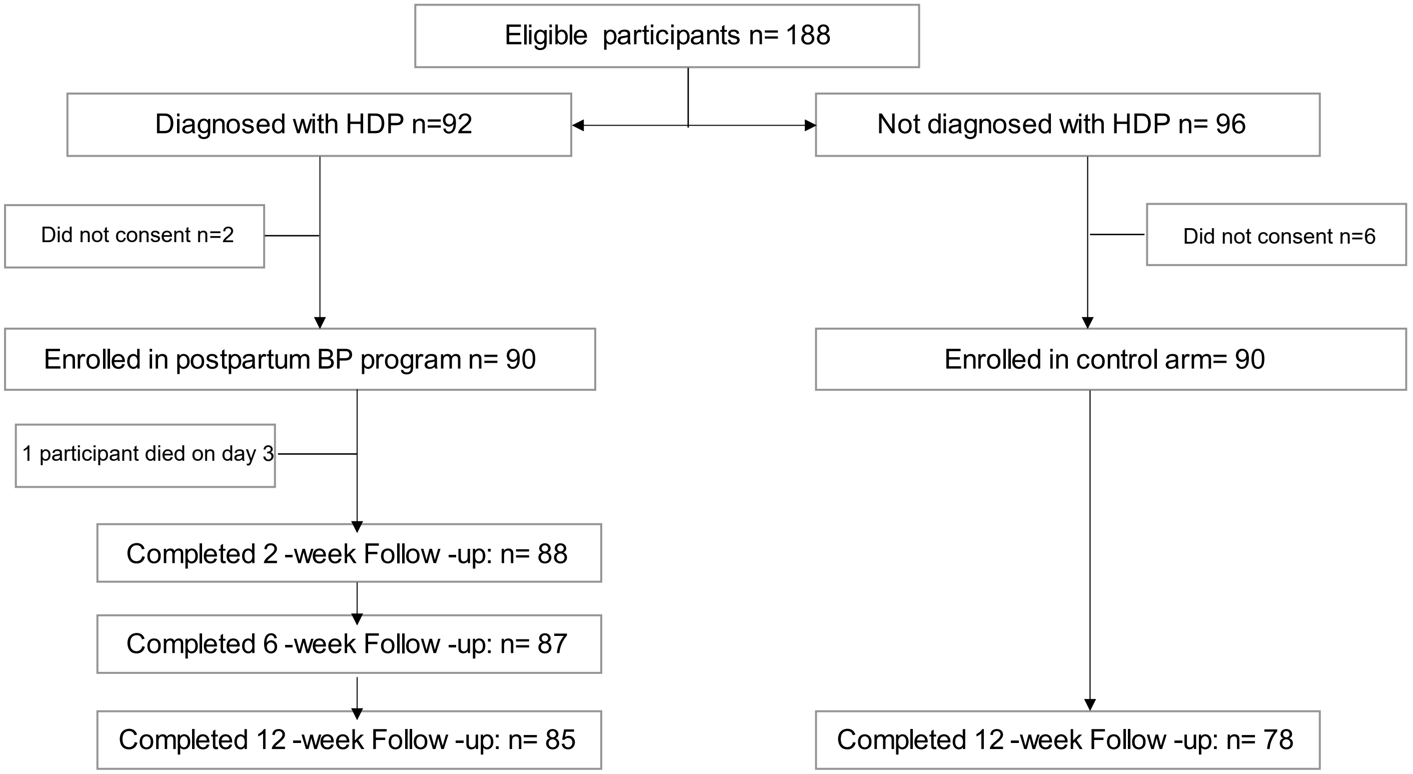

Abbreviations: HDP: hypertensive disorders of pregnancy
